# Editorial: Targeting pulmonary endothelium in acute lung injury and acute respiratory distress syndrome

**DOI:** 10.3389/fcell.2025.1714104

**Published:** 2025-10-08

**Authors:** Lulong Bo, You Shang, Guochang Hu

**Affiliations:** ^1^ Department of Anesthesiology, Changhai Hospital, Naval Medical University, Shanghai, China; ^2^ Department of Critical Care Medicine, Union Hospital, Tongji Medical College, Huazhong University of Science and Technology, Wuhan, Hubei, China; ^3^ Department of Anesthesiology, University of Illinois College of Medicine, Chicago, IL, United States; ^4^ Department of Pharmacology and Regenerative Medicine, University of Illinois College of Medicine, Chicago, IL, United States

**Keywords:** acute lung injury, pulmonary endothelium, biomarker, heparin-binding protein, endothelial barrier function

Acute lung injury (ALI) and acute respiratory distress syndrome (ARDS) remain major causes of mortality in critical care, despite advances in mechanical ventilation and supportive care (Ma et al., 2025; Al-Husinat et al., 2025). The pulmonary endothelium, a thin, semipermeable barrier lining the pulmonary vasculature, plays a central role in the pathogenesis of ALI/ARDS. Endothelial dysfunction drives vascular leak, inflammation, and coagulation abnormalities—hallmark features of ALI/ARDS that culminate in alveolar edema and hypoxemia (Su et al., 2024; Qiao et al., 2024). This Research Topic, “*Targeting Pulmonary Endothelium in Acute Lung Injury and Acute Respiratory Distress Syndrome*,” brings together 13 articles that dissect the molecular mechanisms of endothelial injury, identify novel biomarkers, and explore endothelial-targeted therapeutic strategies, addressing critical gaps in our understanding of these devastating conditions.

A central theme of this Research Topic is elucidating the diverse mechanisms driving endothelial dysfunction in ALI/ARDS, including epigenetic regulation, programmed cell death, and inflammatory signaling crosstalk. Klein underscores the pivotal role of the vascular endothelium in acute pathological conditions, while clarifying the key concepts of endothelial activation, dysfunction, and damage, along with their complex, context-dependent interactions. Cheng et al. highlight the role of histone deacetylases (HDACs)—key epigenetic regulators—in mediating endothelial–mesenchymal interactions during lung fibrosis, a frequent sequela of ARDS. They report that specific HDAC isoforms (*e.g.*, HDAC3, HDAC6) are aberrantly upregulated in pulmonary endothelial cells and fibroblasts from patients with idiopathic pulmonary fibrosis, where they activate transforming growth factor β (TGF-β)/SMAD3 signaling and promote extracellular matrix deposition. Notably, selective HDAC inhibition reduces endothelial-to-mesenchymal transition and extracellular matrix production, underscoring the therapeutic potential of epigenetic modulation in restoring endothelial homeostasis.

Complementing this epigenetic perspective, Han et al. investigate the interplay between the renin–angiotensin system (RAS) and programmed cell death in hyperbaric hyperoxic lung injury. In a rat model of hyperoxic lung injury, they demonstrate that RAS activation triggers necroptosis in type I alveolar epithelial cells, leading to secondary injury of adjacent endothelial cells. Pretreatment with RAS inhibitors reduces angiotensin I expression, suppresses necroptosis-related proteins (receptor interacting protein kinase-1/3, and mixed lineage kinase domain-like protein), and preserves endothelial barrier integrity, thereby establishing a mechanistic link between RAS-mediated cell death and endothelial dysfunction.

Xiao et al. further advance our understanding of cell death pathways by focusing on caspase-mediated pyroptosis, apoptosis, and necroptosis in ALI. Their review highlights caspase-11 (in murine) and caspase-4/5 (in humans) as critical mediators of endothelial pyroptosis in response to lipopolysaccharide (LPS). Upon activation, these caspases cleave gasdermin D (GSDMD), leading to the formation of membrane pores that compromise endothelial barrier integrity. In contrast, caspase inhibitors (*e.g.*, Ac-FLTD-CMK) or GSDMD-targeted agents (*e.g.*, disulfiram) have been shown to preserve endothelial barrier function. Complementing this, Yao et al. systematically review therapeutic strategies targeting ferroptosis in the management of ARDS, emphasizing the use of antioxidants, glutathione peroxidase 4 activators, iron chelators, and lipid peroxidation inhibitors. These interventions aim to mitigate oxidative stress overload, restore the endogenous antioxidant defense system of pulmonary cells, and inhibit iron-mediated ferroptotic cell death, thereby preserving alveolar-endothelial barrier function.

Accurate diagnosis and prognosis of ALI/ARDS remain challenging; however, studies in this Research Topic identify endothelial-derived molecules as promising biomarkers. Liu et al. investigate heparin-binding protein—a neutrophil-derived inflammatory mediator—in bronchoalveolar lavage fluid and plasma of ARDS patients. Their animal and human studies demonstrate that heparin-binding protein levels in bronchoalveolar lavage fluid are significantly elevated in ARDS compared with cardiogenic pulmonary edema and show a stronger correlation with lung injury severity than plasma heparin-binding protein. Xiao et al. further identify CMTM3 (CKLF-like MARVEL transmembrane domain-containing 3), as both a biomarker and therapeutic target. Using LPS- or hypoxia/reoxygenation-stimulated human umbilical vein endothelial cells, they demonstrate that CMTM3 expression is upregulated in injured endothelial cells, enhancing vascular permeability and interleukin (IL)-6/tissue necrosis factor (TNF)-α production. In CMTM3 knockout mice with ARDS, pulmonary vascular permeability and lung injury scores are reduced, while survival is improved—establishing CMTM3 as a critical mediator of endothelial dysfunction with diagnostic and therapeutic potential.

Translational studies in this Research Topic bridge preclinical findings to clinical practice, highlighting strategies to restore endothelial function in ALI/ARDS. Xu et al. perform a network meta-analysis of 26 clinical trials (5,071 patients) to compare the efficacy of corticosteroids, neuromuscular blocking agents, and inhaled nitric oxide—three commonly used interventions—on ARDS outcomes related to endothelial function. Their analysis revels that dexamethasone reduces new infection events (a surrogate for endothelial barrier integrity) and increases ventilator-free days, while vecuronium bromide decreases 28-day mortality, likely by reducing endothelial inflammation and oxidative stress.

In addition, studies examining other key mechanisms of endothelial dysfunction in ALI/ARDS further enhance the scope of this Research Topic. For instance, review by Xu et al. focuses on specific signaling pathways, such as phosphatidylinositol 3-kinase (PI3K)/AKT and Notch1 show that their aberrant activation disrupts endothelial junctions and increases vascular permeability. In preclinical ALI models, pharmacologic inhibition of these overactivated pathways restores endothelial integrity, offering additional therapeutic avenues.

Heat stroke is a life-threatening condition with increasing incidence and mortality driven by global warming. Wang et al. review recent advances in understanding the pathogenesis of heat stroke-induced endothelial injury, including glycocalyx degradation and cell death activation and discuss potential therapeutic approaches. They emphasize the central role of the endothelium and highlight related biomarkers with diagnostic and prognostic value.

Although the studies in this Research Topic advance our understanding of endothelial targeting in ALI/ARDS, several critical gaps remain. First, most preclinical studies rely on homogeneous endothelial cell populations or animal models that fail to recapitulate the heterogeneity of human pulmonary endothelium (Mora et al., 2025). As Yang et al. review, future work should harness single-cell RNA sequencing to delineate subtype-specific endothelial responses. Second, the long-term outcomes of endothelial-targeted therapies, such as fibrosis, and vascular remodeling, remain poorly studied; longitudinal studies are needed to determine whether restoring endothelial function reduces late-stage ARDS sequelae (Cusack et al., 2023). Finally, clinical translation of preclinical findings is limited by the absence of endothelial-specific drug delivery systems. Nanoparticle-based targeting of endothelial markers holds promise for enhancing both the efficacy and safety of existing therapies.

In conclusion, this Research Topic underscores the pulmonary endothelium as a central player in ALI/ARDS pathophysiology and a promising therapeutic target. The articles herein unravel epigenetic, cell death, and inflammatory mechanisms of endothelial dysfunction, validate novel biomarkers, and confirm the efficacy of endothelial-targeted therapies. By bridging basic and translational research, this Research Topic offers a roadmap for advancing precision medicine strategies aimed at restoring endothelial homeostasis and improving outcomes in ALI/ARDS.
